# Galanin 2 Receptor: A Novel Target for a Subset of Pancreatic Ductal Adenocarcinoma

**DOI:** 10.3390/ijms241210193

**Published:** 2023-06-15

**Authors:** Pawel Namsolleck, Barbara Kofler, Gert N. Moll

**Affiliations:** 1 Lanthio Pharma, 9727 DL Groningen, The Netherlands; pawel.namsolleck@gmail.com; 2 PCDA Pharma Consulting & Data Analytics, 9791 CH Ten Boer, The Netherlands; 3 Research Program for Receptor Biochemistry and Tumor Metabolism, Department of Pediatrics, University Hospital of the Paracelsus Medical University, Muellner Hauptstr. 48, 5020 Salzburg, Austria; 4 Department of Molecular Genetics, Groningen Biomolecular Sciences and Biotechnology Institute, University of Groningen, Linnaeusborg, Nijenborg 7, 9747 AG Groningen, The Netherlands

**Keywords:** galanin, galanin receptor, pancreatic ductal adenocarcinoma, lanthi-galanin, targeted treatment

## Abstract

Galanin is a 30 amino acid peptide that stimulates three subtype receptors (GAL_1–3_R). M89b is a lanthionine-stabilized, C-terminally truncated galanin analog that specifically stimulates GAL_2_R. We investigated the potential of M89b as a therapeutic for pancreatic ductal adenocarcinoma (PDAC) and assessed its safety. The anti-tumor activity of subcutaneously injected M89b on the growth of patient-derived xenografts of PDAC (PDAC–PDX) in mice was investigated. In addition, the safety of M89b was assessed in vitro using a multi-target panel to measure the off-target binding and modulation of enzyme activities. In a PDAC–PDX with a high *GAL_2_R* expression, M89b completely inhibited the growth of the tumor (*p* < 0.001), while in two PDAC–PDXs with low *GAL_2_R* expression, low or negligeable inhibition of tumor growth was measured, and in the PDX without *GAL_2_R* expression no influence on the tumor growth was observed. The M89b treatment of the *GAL_2_R* high-PDAC–PDX-bearing mice led to a reduction in the expression of *RacGap1* (*p* < 0.05), *PCNA* (*p* < 0.01), and *MMP13* (*p* < 0.05). In vitro studies involving a multi-target panel of pharmacologically relevant targets revealedexcellent safety of M89b. Our data indicated that GAL_2_R is a safe and valuable target for treating PDACs with high *GAL_2_R* expression.

## 1. Introduction

Pancreatic ductal adenocarcinoma (PDAC) is a lethal disease with a poor prognosis. PDAC accounts for more than 90% of all pancreatic cancers [1,2]. It is the seventh leading cause of cancer-related death globally, and is ranked third in the United States [1]. A prediction published in 2014 states that pancreatic cancers will be the second leading cause of cancer-related mortality before 2030 [3,4].

Several factors, which are associated with a higher incidence of PDAC, have been identified [5]. The lack of screening and the prevalence of early metastasis contribute to the high mortality rate of pancreatic cancer [6]. Furthermore, the dense tumoral stroma, a distinct feature of PDAC, constitutes a barrier for therapeutics. The limited destruction of the stroma may enhance the accessibility of the tumor, but on the other hand, may also promote metastasis [5]. Nearly 80% of PDAC patients who undergo surgery have disease recurrence [7]. Most PDACs contain one or more of the four main driver mutations: the Kirsten rat sarcoma viral oncogene homolog (*KRAS*), the cyclin-dependent kinase inhibitor 2A (*CDKN2A*), the tumor suppressor protein 53 (*TP53*), or the small mothers against decapentaplegic homolog 4 (*SMAD4*) [8]. The incidence of the *KRAS* mutations in PDAC is approximately 86% [9]. Barriers in the efficacy and adverse effects of the current therapies for this unmet medical need have been reviewed [10]. A recent clinical phase 3 study of liposomal irinotecan plus 5-fluorouracil/leucovorin plus oxaliplatin showed significant improvement versus nab-paclitaxel plus gemcitabine in patients with PDAC [11]. In view of the lethality of the disease, and in view of the lack of efficacy and the harshness of the existing treatments, more effective treatments are urgently needed.

Human galanin binds to three class-A, G-protein coupled receptors: GAL_1_R, GAL_2_R, and GAL_3_R [12]. Galanin-positive nerve fibers have been identified in the pancreas of different species [13,14]. While galanin receptors are abundantly expressed in mouse islets, they are present only at low levels in human islets [15]. Furthermore, galanin displays strong inhibitory effects toward insulin secretion [16].

The galaninergic system is also involved in tumorigenesis, the invasion/migration of tumor cells and angiogenesis, and has been correlated with the tumor stage, proliferation, metastasis, and recurrence rate [17,18]. For example, in head and neck cancer, GAL_2_R shows a tumor suppressor activity [19]. The inactivation of GAL_2_R by promoter hypermethylation decreased disease-free survival [19]. GAL_2_R strongly exerted anti-tumor activity via two important mechanisms: (1) tumor cell cycle arrest mediated via the extracellular-regulated protein kinase-1/2 (ERK1/2)-dependent effects on the cyclin-dependent kinase inhibitors (CKI) and cyclin D1, and (2) induction of caspase-3-dependent apoptosis [20]. In contrast, GAL_1_R only exerted the above-mentioned tumor cell cycle effect without inducing apoptosis. GAL_3_R, on the other hand, was not considered a tumor suppressor [20]. Interestingly, subcutaneous injection of galanin reduced the number of chemically induced PDACs in rats [21]. A triple treatment of PDAC xenografts with octreotide (a somatostatin analogue), serotonin, and galanin increased the survival of mice, albeit the proliferation index and the labelling index for the epidermal growth factor (EGF) increased significantly compared to the vehicle-treated controls [22]. GAL_2_R appears to be therapeutically relevant in PDAC as it is expressed in the pancreas [23] and pancreatic cancers [24]. Of note, there appears to be a trend for longer survival in patients with a higher expression of GAL_2_R in the PDAC [25]. Therefore, selectively stimulating GAL_2_R could be a valuable target for PDAC therapy. PDACs are shielded by fibrotic tissue, which might limit drug access to the tumor. Thus, an additional rationale for stimulating GAL_2_R was based on its anti-fibrotic activity [26]. The GAL_2_R stimulation-mediated anti-fibrotic activity might enhance the accessibility of the tumor for a GAL_2_R agonist and, concomitantly, for other small drugs to the tumor. The fibrosis and collagen around PDAC in a patient-derived xenograft (PDX) model in mice was similar to the fibrosis observed around PDACs in humans. PDX tumors contain α-smooth muscle actin-expressing fibroblasts similar to patient tumors. The collagen structures in the PDX models of PDAC closely resemble those found in the original tumor [27]. PDX models have great predictive value for clinical outcomes [28].

A lanthionine imposed conformational constraint can enhance the target specificity of a peptide [29,30] and locally protects against breakdown by peptidases [31]. Resistance to breakdown by peptidases may prolong the in vivo half-life (T_1/2_). A lanthionine-stabilized angiotensin analog had a T_1/2_ of 2.1 to 2.6 h [32] in humans, whereas a linear angiotensin was broken down within a minute [33]. Lanthionine introduction in a peptide may favor membrane translocation and extend the options for delivery routes [34]. M89b is a stable lanthionine-constrained galanin analog that stimulates GAL_2_R. In a previous study, M89b showed a promising combination of efficacy, specificity, and safety [30]. The aim of the present study was to elucidate whether the GAL_2_R-selective agonist M89b is a safe compound withanti-PDAC activity.

## 2. Results

### 2.1. Treatment of PDAC–PDX Using M89b

The expression analysis revealed that *GAL_1_R* was not expressed in any of the four PDAC–PDX cell lines, and *GAL_3_R* was only detectable in Panc11074. The *GAL_2_R* expression in the four PDXs ranged from a high expression (Panc11074), a low expression (Panc10056), a very low expression (Panc11495), to no expression at all (Panc9759) (Table 1).

**Figure 1:**
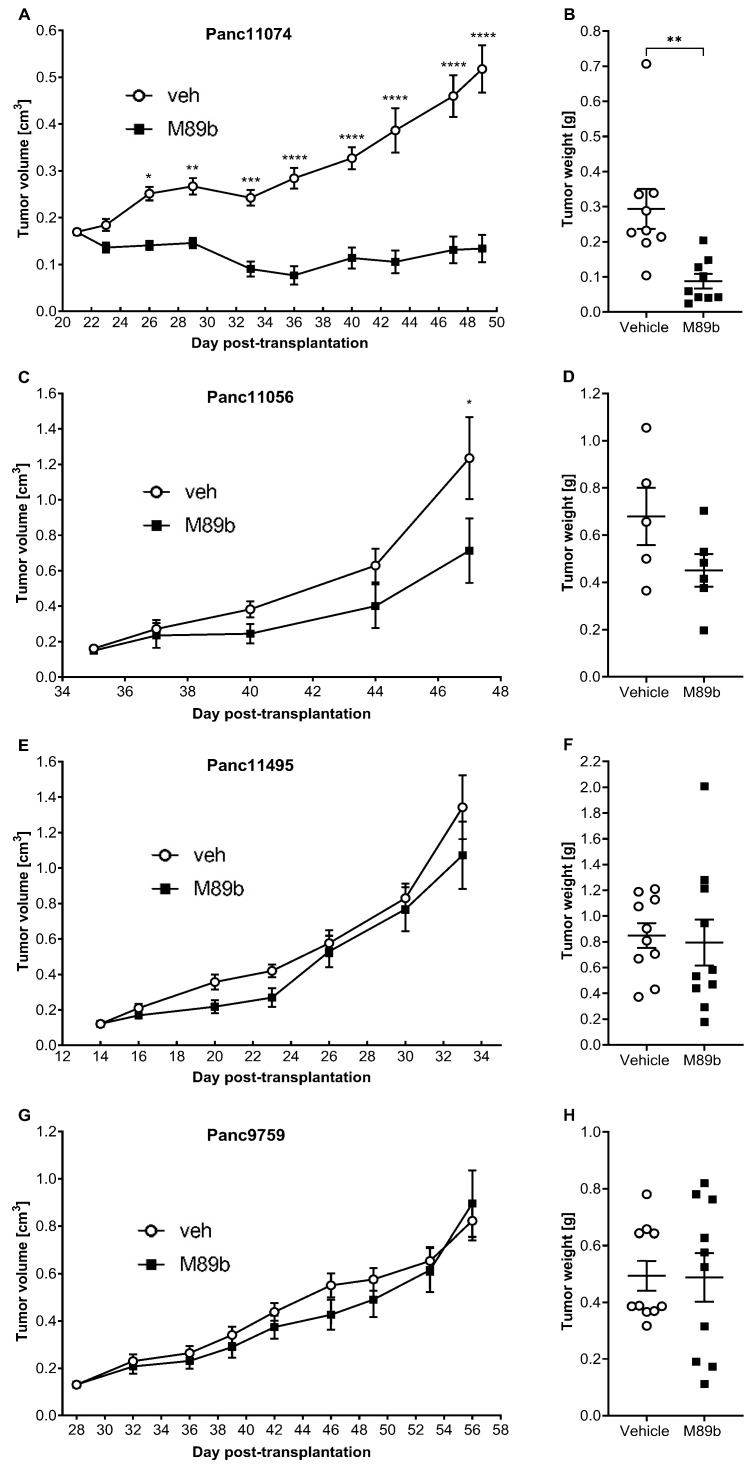
Effect of M89b treatment on the proliferation (**A**,**C**,**E**,**G**) and tumor weight (**B**,**D**,**F**,**H**) of PDAC–PDX: (**A**,**B**) Panc11074, (**C**,**D**) Panc11495, (**E**,**F**) Panc11056, and (**G**,**H**) Panc9759. The tumor weight was determined on the termination day. A two-way repeated measures ANOVA using Sidak’s multiple comparisons post-test was used for the statistical analysis of the tumor growth curves. A Mann–Whitney U test was used to examine the significant differences in the tumor weight of the mice treated with M89b compared to the vehicle controls. * *p* < 0.05, ** *p* < 0.01, *** *p* < 0.001, **** *p* < 0.0001, *n* = 5–9.

Next, we generated PDAC–PDXs and treated mice with M89b. Subcutaneously administered M89b (70 µg/kg) completely inhibited the growth of the *GAL_2_R* high-Panc11074–PDX (Figure 1A). This strong anti-tumor effect was reflected by the low T/C value of 25.9% at study day 49 and was also mirrored by a three-fold reduced tumor weight in the M89b-treated animals compared to the vehicle-treated controls (0.088 ± 0.020 g mean tumor weight for the M89b-treated group, versus 0.294 ± 0.057 g for the vehicle-treated group, *p* < 0.001; Figure 1B).

For the PDAC–PDXs with a lower or absent *GAL_2_R* expression, the M89b treatment had a minor or no effect. The treatment of Panc10056 with M89b showed a lower tumor progression, but a statistical significance (*p* < 0.05) was only reached at the latest time point (Figure 1C), which was also reflected by a lower tumor weight compared to the vehicle treatment (*p* = 0.1477; Figure 1D). For Panc11495 and Panc9759, the M89b treatment had no effect on the tumor growth (Figure 1E,G) or the tumor weight (Figure 1F,H).

### 2.2. Effect of M89b Treatment on the Markers of Tumor Progression

**Figure 2:**
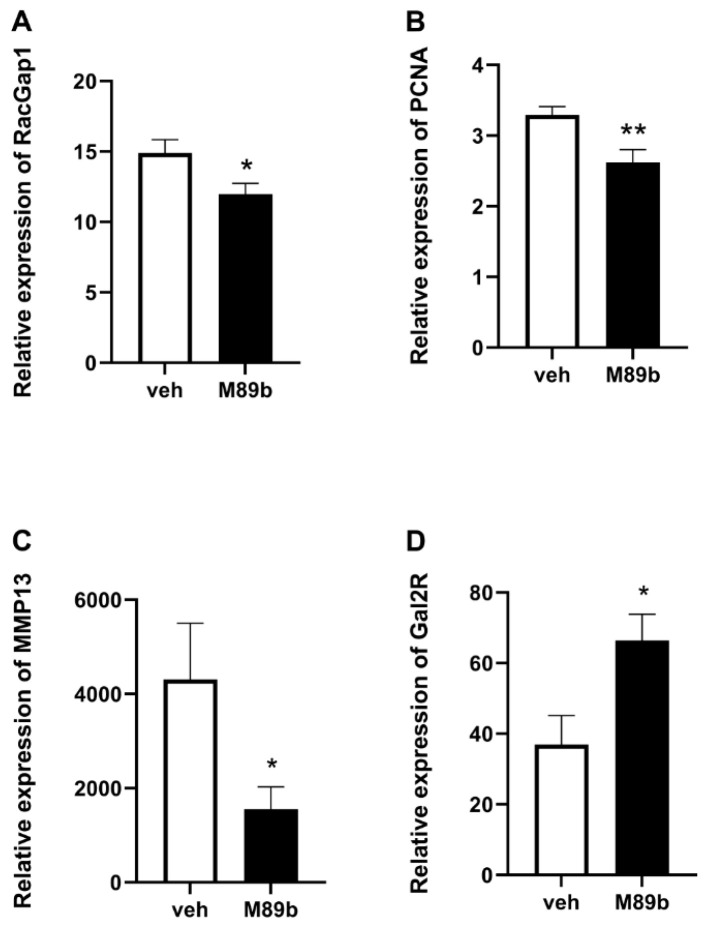
Treatment with M89b reduced the expression of *RacGap1* (**A**), *PCNA* (**B**), and *MMP13* (**C**) and enhanced the expression of *GAL_2_R* (**D**) in the Panc11074 PDX tissues. The PCR analysis was performed in quadruplicate of each PDX. The data are presented ± SEM (*n* = 9). The significance was tested as indicated in the Material and Methods. * *p* < 0.05. ** *p* < 0.01 compared to the vehicle-treated controls.

The M89b treatment on the Panc11074 PDX-bearing mice decreased the expression of the proliferation-inducing genes, including the tumor proto-oncogene Rac GTPase-activating protein 1 (*RacGap1*; *p* < 0.05), the cell nuclear antigen (*PCNA*; *p* < 0.01), and the matrix metalloproteinase 13 (*MMP13*; *p* < 0.05) in the tumor tissue (Figure 2A–C). On the other hand, the M89b treatment of Panc11074 PDX had no effect on the expression of the apoptosis regulators *BCL-2* and *Bax*, the markers of proliferation *MKI67* and topoisomerase II alpha (*TOP2A*), and the tumor suppressor *TP53* (Figure S1A–E). Interestingly, the M89b treatment upregulated *GAL_2_R* in the Panc11074 PDX (*p* < 0.05; Figure 2D). The upregulation of GAL_2_R, either by endogenous galanin and/or by M89b, might have contributed, in part, to the anti-tumor effects of M89b.

### 2.3. In Vitro and In Vivo Safety

In an in vitro safety pharmacology profiling panel (Eurofins-Cerep), which is commonly used for the early screening of off-target binding, including 70 pharmacologically relevant targets, an excess of M89b (1 µM) did not reveal any significant binding competition (>50% inhibition) with the picomolar to nanomolar concentrations of the specific radiolabeled target binders for any of the tested targets (Figure S2). Similarly, M89b (>50% effect) did not interfere with any of the tested 25 enzyme and uptake assays (Figures S3–S5), with the exception of histone deacetylase 6 (HDAC6) (Figure S3). Indeed, the inhibition of HDAC6 is thought to beneficially contribute to the treatment of several types of cancer [35]. Hence, rather than a safety concern, the M89b-mediated inhibition of HDAC6 could have a therapeutic efficacy in PDAC. However, since M89b has no anti-tumor activity in a PDAC–PDX without *GAL_2_R* expression (Figure 1E–H), it appeared that either M89b failed to translocate across the cellular and nuclear membranes to reach HDAC6 in tumor cells or the inhibition of HDAC6 by M89b in vivo was incapable of yielding an anti-tumor effect. Adding to its safety profile, no M89b-induced adverse effects were observed in vivo, which was supported by observations of stable animal body weights that were indistinguishable from the weights of the vehicle-treated animals (Figure S6).

## 3. Discussion

Here, we provide the first evidence that a GAL_2_R-specific stable agonist could inhibit growth of PDACs (Figure 1), which present with sufficient *GAL_2_R* expression (Table 1). Our study was consistent with earlier studies demonstrating that galanin reduced the number of chemically induced PDACs, and that a combination treatment of galanin with octreotide and serotonin increased the survival of PDAC xenograft-bearing mice [21,22]. Neither of these two studies investigated which of the galanin receptors might be responsible for the observed tumor-suppressive effects in these PDAC mouse models. A recent study reported pro-tumor effects of galanin in head and neck cancer via the activation of PBMCs inducing immune-suppressive and pro-tumoral effects [36]. Notwithstanding the fact that we used an immunocompromised mouse model, the work by Iishi et al. [20] similarly used an immunocompetent model. Therefore, we do not expect an immunosuppressive effect of galanin agonism in PDAC treatment. A trend for longer survival in patients with a higher expression of GAL_2_R in the PDAC was observed [25]. M89b enhanced the expression of *GAL_2_R* (Figure 2D). A wide range of patient samples needs to be subjected to immunohistochemistry, western blot, and RNA analysis in a follow-up study.

Consistent with its high specificity, M89b also appeared to have excellent safety. No adverse effects of M89b were observed in this study on subcutaneously delivered M89b, as well as in our previous study investigating intranasally delivered M89b [30]. In view of its mode of action, excellent specificity, and safety, stimulating GAL_2_R is completely distinct from all the current therapeutic approaches for treating PDAC. We, therefore, suspect that synergism with existing therapies is likely. As a consequence, future positioning should not be ruled out in combination therapy, maintenance therapy, or palliative therapy. Taken together, the current literature and experimental data underpin the hypothesis of exceptional perspectives for the selective stimulation of GAL_2_R in the treatment of patients with PDAC, which is an urgent unmet medical need. The identification of patients with GAL_2_R-expressing PDACs could be performed either by RT-PCR or immunohistochemical staining of the tumor tissue for the GAL_2_R expression or using GAL_2_R-selective PET imaging. For example [(18)F]-lanthionine-bombesin analogs were discovered for GRPR-positive tumor imaging of prostate cancer xenografts [37]. Patients with PDACs presenting a medium to high *GAL_2_R* expression could subsequently be treated using GAL_2_R agonists. The exact cut off level of the GAL_2_R expression, which will be necessary for a therapeutic response to a GAL_2_R agonist, needs be determined in follow-up studies.

In conclusion, targeting GAL_2_R-expressing PDACs using selective agonistic lanthi-galanin peptides is a novel approach that ha therapeutic potential for a subset of PDAC patients, representing an opportunity for the further development of personalized medical therapy.

## 4. Materials and Methods

### 4.1. Animal Maintenance and Treatment

For all the in vivo studies, animal welfare was maintained in strict accordance with the principles governing the use of animals in experiments from the European Communities and German legislation [38]. These studies were approved by the local responsible authorities in Berlin, Germany (Approval No. A 0010/19, LaGeSo Berlin, Germany). The PDAC–PDX models used in this study were performed by EPO Berlin. Immunodeficient NMRI nu/nu female mice (EPO GmbH) at ages of 6 to 8 weeks were used. Female mice were used since these were considered to be less aggressive in PDX studies and more viable than male mice [39]. The PDAC–PDXs (Table 1) were inoculated subcutaneously (s.c.) outside the tumor, and the tumors were grown to achieve a palpable tumor size prior to the start of the respective treatments. The treatment using M89b began with palpable tumors after the randomization of the animals with respect to the tumor volumes. The GAL_2_R agonist M89b was solved in sterile PBS and was applied s.c. via daily injections with a 70 µg/kg dose for a maximum of 59 days (or earlier, as defined by the humane end point for the study). The growth inhibitory activity of the compound M89b was evaluated by the determination of the tumor volumes (TV). During the study, the tumor volumes and body weights were measured twice weekly, and the ratio of the mean tumor volumes between the M89b-treated group and the vehicle-treated group (T/C) was calculated. The tumor volume and T/C were calculated according to the following equations.

Tumor volume = [(tumor width) × (tumor width) × (tumor length)] ÷ 2

T/C = T ÷ C

T: Mean estimated tumor volume of the compound-treated group.

C: Mean estimated tumor volume of the vehicle-treated group.

The tumors were collected after the termination of anesthetized mice and the tumor weight was determined. The tumor samples were snap-frozen for subsequent gene expression analysis.

### 4.2. mRNA Expression Analysis

The mRNA was isolated from the PDX tissue (approximately 20 mg) using the Rneasy kit (Qiagen, Germantown, MD, USA), following instructions of the manufacturer. The synthesis of cDNA was performed using 7 µg of the RNA SuperScript™ IV VILO™ Master Mix with ezDNase™ Enzyme (Thermo Fisher, Waltham, MA, USA). A duplex quantitative PCR was performed using primer/probes from Thermo Fischer (Table S1) and the Advanced Master Mix (Thermo Fisher). The PCR program involved the following steps: 2 min at 50 °C, 40×, 1 s at 95 °C, and 20 s at 60 °C.

### 4.3. In Vitro Safety Studies

The multi-target safety panel study was executed by Eurofins Cerep, 86600 Celle Levescault, France. M89b was tested at 1 µM. The M89b target binding was calculated as a percentage of the inhibition of the binding of a radioactively labeled ligand that was specific for each target. The M89b-mediated enzyme inhibition was calculated as a percentage of the inhibition of the control enzyme activity. The results showing an inhibition or a stimulation greater than 50% were considered to represent the significant effects of M89b. Depending on the target and the reference compound, a final concentration of the reference compound and time of incubation was followed. In all the cases, the concentration of the reference compound was several orders of magnitude lower than 1 µM. The in vitro binding study conditions are provided in Tables S2 and S3. The in vitro enzyme and uptake assay conditions are provided in Table S4.

### 4.4. Statistical Analyses

For the statistical analysis of the tumor volume (Figure 1) over time, depending on the time and treatment, a repeated measures ANOVA with Sidak’s multiple comparisons post-test for selecting the sphericity was used. Since there were no missing values, a repeated measures ANOVA was used. A full model was applied to the test column, row, and column/row interaction effect. The repeated measures test was more powerful because it separated the between-subject variability from the within-subject variability. For the statistical analysis of the tumor weight, an unpaired two-tailed *t*-test was used. Since the SDs were not equal, the Welch’s correction was applied. Due to an insufficient n for the Panc11056 tests, the normality could not be conducted. Thus, the tumor weight data were additionally analyzed using a non-parametric Mann–Whitney test. The results followed the same pattern as the *t*-test except for Panc11074, where the significance increased from *p* = 0.0037 for the *t*-test to *p* = 0.0005 for the Mann–Whitney test.

For the qPCR data analyses (Figure 2), four normality tests were performed first (D’Agostino and Pearson, Anderson–Darling, Shapiro–Wilk, and Kolmogorov–Smirnov). The data regarding *PCNA* passed all the normality tests, and therefore, an unpaired two-tailed *t*-test was applied. The data from *RacGap1*, *MMP13*, and *GAL_2_R* were non-parametrically analyzed.

A *p*-value < 0.05 was considered statistically significant. For the safety assays of target-binding and the modulation of the enzyme activity, effects > 50% were considered significant.

## Figures and Tables

**Table 1:** Genetic alterations and galanin receptor expressions in human pancreatic ductal adenocarcinoma PDX models. RNA seq. data provided by EPO GmbH. *KDR*: kinase insert domain receptor gene; *SRC*: SRC proto-oncogene.

PDX	Mutation Status	*GAL_1_R*	*GAL_2_R*	*GAL_3_R*
Panc11074	*KDR*, *KRAS*, *SMAD4*, *CTNNB*, *TP53*	0.0	16.7	0.1
Panc11056	*KRAS*	0.0	0.7	0.0
Panc11495	*KRAS*	0.0	0.3	0.0
Panc9759	*KDR*, *KRAS*, *SMAD4*, *SRC*, *TP53*	0.0	0.0	0.0

## Data Availability

The data will be readily available upon request from the corresponding authors.

## Supplementary Materials

The supporting information can be downloaded at: https://www.mdpi.com/article/10.3390/ijms241210193/s1.
