# Supplementary material for: Galanin 2 Receptor: A Novel Target for a Subset of Pancreatic Ductal Adenocarcinoma
Source: Int J Mol Sci. 2023 Jun 15;24(12):10193. doi: 10.3390/ijms241210193 (PMC10299363; doi:10.3390/ijms241210193)
Supplement: Supplementary file 1 [file ijms-24-10193-s001.zip › ijms-2420079-supplementary.pdf]

SUPPLEMENTARY DATA

Galanin 2 receptor: a novel target in a subset of pancreatic ductal adenocarcinoma

Pawel Namsolleck, Barbara Kofler, Gert N. Moll

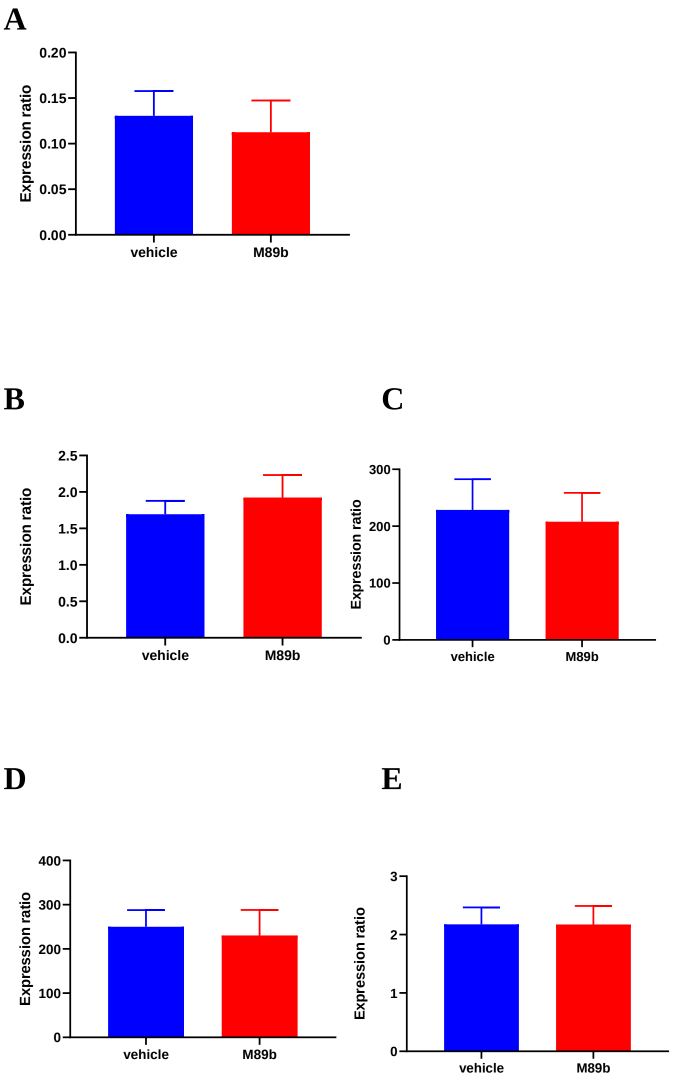

Figure S1. Relative expression of markers after treatment with vehicle or M89b.

Treatment of mice with M89b had no effect on expression of *Bcl2* (**A**), *MKI67* (**B**), *Bax* (**C**), *TOP2A* (**D**) and *TP53* (**E**) in Panc11074 PDX tissue. Data are presented ± SEM, (n=9).

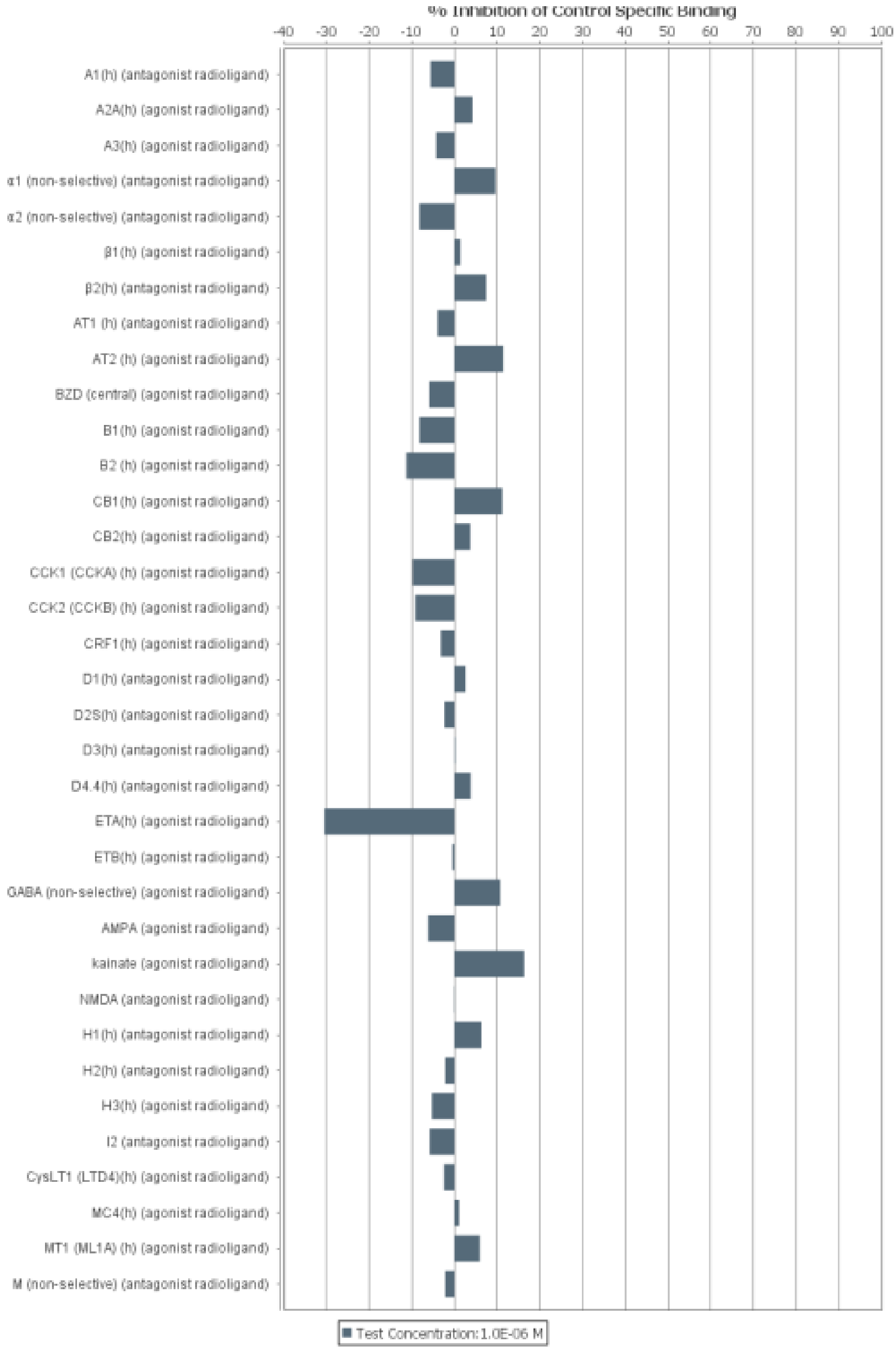
% Inhibition of Control Specific Binding
-40
-30
-20
-10
0
10
20
30
40
50
60
70
80
90
100
A1(h) (antagonist radioligand)
A2A(h) (agonist radioligand)
A3(h) (agonist radioligand)
α1 (non-selective) (antagonist radioligand)
α2 (non-selective) (antagonist radioligand)
β1(h) (agonist radioligand)
β2(h) (antagonist radioligand)
AT1 (h) (antagonist radioligand)
AT2 (h) (agonist radioligand)
BZD (central) (agonist radioligand)
B1(h) (agonist radioligand)
B2 (h) (agonist radioligand)
CB1(h) (agonist radioligand)
CB2(h) (agonist radioligand)
CCK1 (CCKA) (h) (agonist radioligand)
CCK2 (CCKB) (h) (agonist radioligand)
CRF1(h) (agonist radioligand)
D1(h) (antagonist radioligand)
D2S(h) (antagonist radioligand)
D3(h) (antagonist radioligand)
D4.4(h) (antagonist radioligand)
ETA(h) (agonist radioligand)
ETB(h) (agonist radioligand)
GABA (non-selective) (agonist radioligand)
AMPA (agonist radioligand)
kainate (agonist radioligand)
NMDA (antagonist radioligand)
H1(h) (antagonist radioligand)
H2(h) (antagonist radioligand)
H3(h) (agonist radioligand)
I2 (antagonist radioligand)
CysLT1 (LTD4)(h) (agonist radioligand)
MC4(h) (agonist radioligand)
MT1 (ML1A) (h) (agonist radioligand)
M (non-selective) (antagonist radioligand)
Test Concentration: 1.0E-06 M

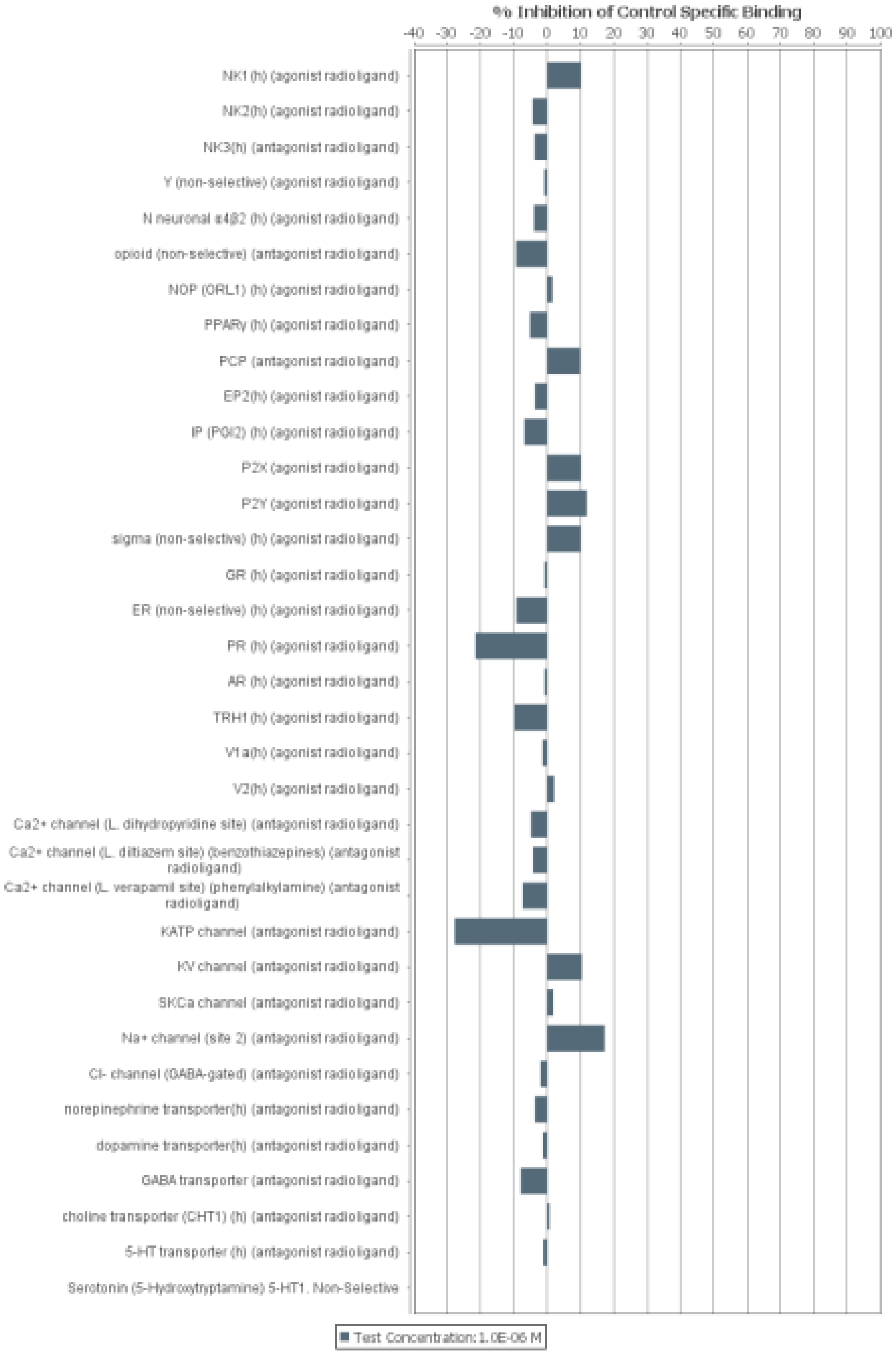

Figure S2. Target binding at 1 µM M89b in a multi-target safety panel.

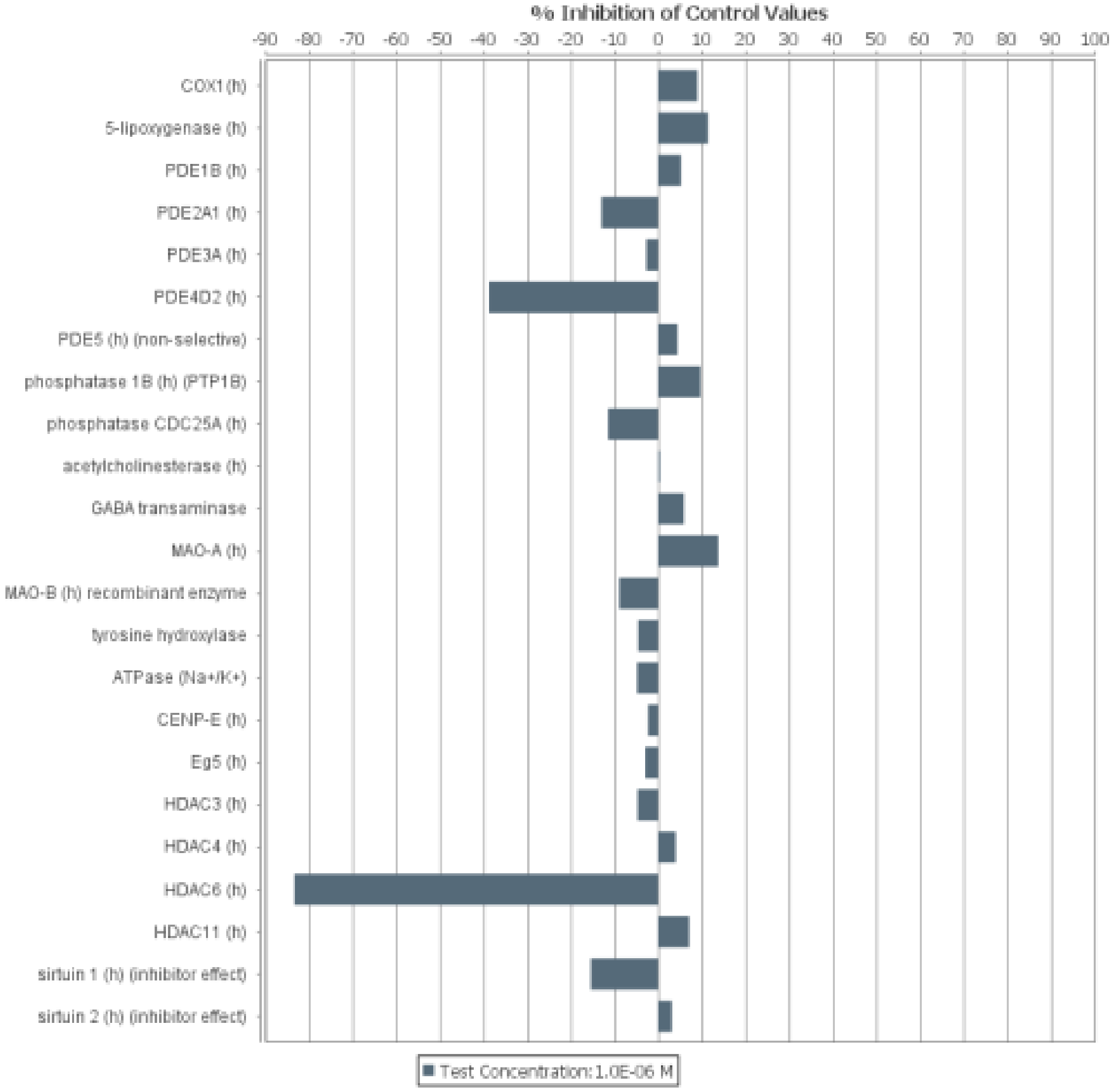

Figure S3. Enzyme and uptake assays at 1 µM M89b.

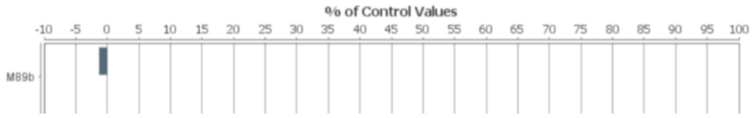

Figure S4. Activator effect of M89b on adenylyl cyclase.

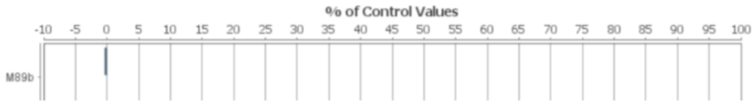

Figure S5. Activator effect of M89b on guanyl cyclase.

A

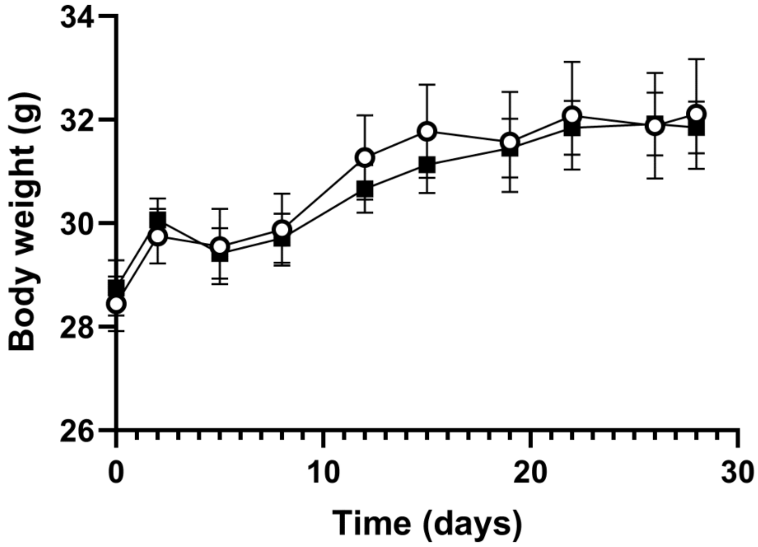
34
32
30
28
26
Body weight (g)
0
10
20
30
Time (days)

B

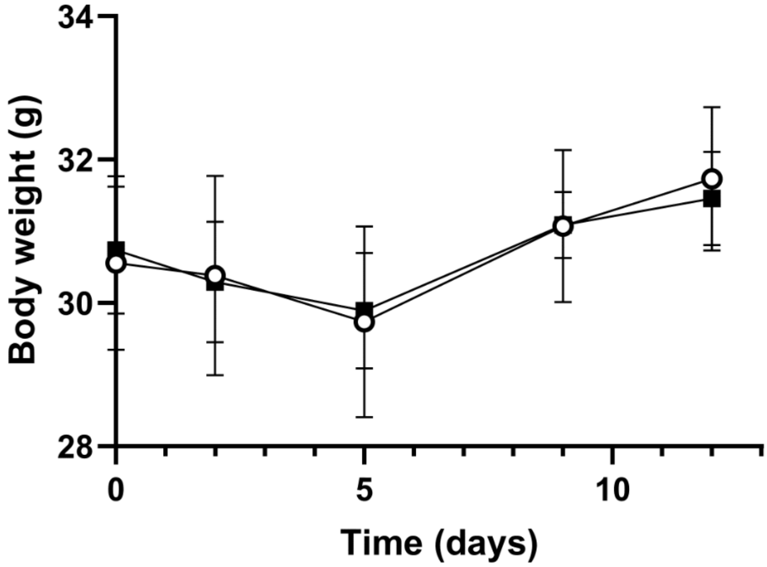
34
32
30
28
Body weight (g)
0
5
10
Time (days)

C

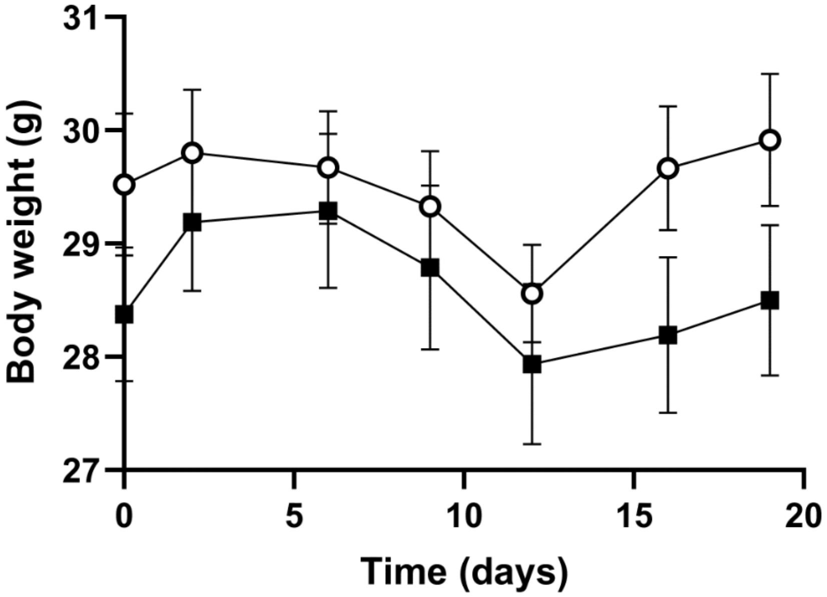

D

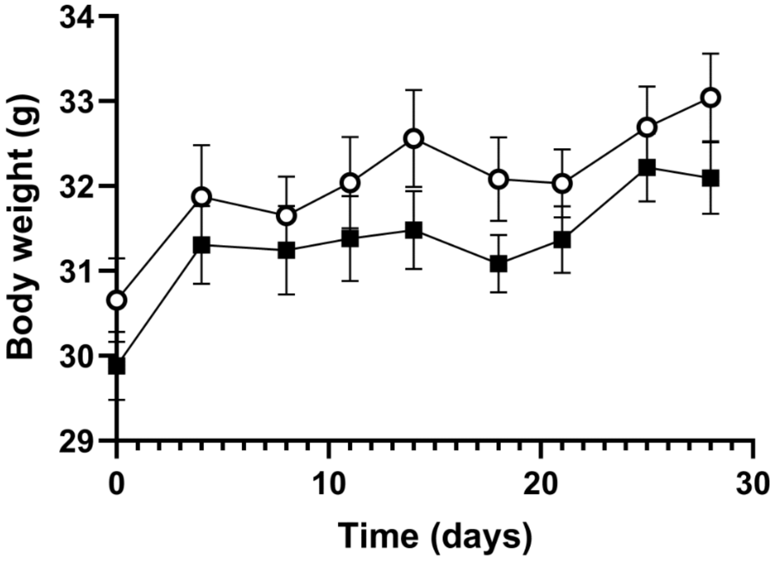

Figure S6ABCD. Effect of M89b treatment on body weight of mice with PDX xenografts (A): Panc11074, (B): Panc11056, (C): Panc11495, (D): Panc9759 (n=5-9 in each group).

**Table S1. Probes.**	
**Probe**	**Lot Nr**
GalR2 Hs00605839_m1	1672852 + 1439439
MKI67 Hs01032433_m1	1782541
PCNA Hs00427214_g1	P200316-008-H07
RacGAP1 Hs01100049_mH	1713072
TOP2A Hs01032137_m1	P200312-009-H08
BCL2 Hs01048932_g1	P200918-007-H01
TP53 Hs01034249_m1	1769968
Bax Hs00180269_m1	P200312-009-H10
MMP13 Hs00942584_m1	1759123

Table S2. *In vitro* target binding conditions.

Assay	Source	Ligand	Conc.	Kd	Non Specific	Incubation	Detection Method	Bibl.
**Receptors**								
**A_1 *(h)* (antagonist radioligand)**	human recombinant (CHO cells)	[^{3}H]DPCPX	1 nM	1.7 nM	DPCPX (1 µM)	60 min RT	Scintillation counting	245
**A_{2A} *(h)* (agonist radioligand)**	human recombinant (HEK-293 cells)	[^{3}H]CGS 21680	6 nM	27 nM	NECA (10 µM)	120 min RT	Scintillation counting	141
**A_3 *(h)* (agonist radioligand)**	human recombinant (HEK-293 cells)	[^{125}I]AB-MECA	0.15 nM	0.22 nM	IB-MECA (1 µM)	120 min RT	Scintillation counting	206
**α_1 (non-selective) (antagonist radioligand)**	rat cerebral cortex	[^{3}H]prazosin	0.25 nM	0.09 nM	prazosin (0.5 µM)	60 min RT	Scintillation counting	88
**α_2 (non-selective) (antagonist radioligand)**	rat cerebral cortex	[^{3}H]RX 821002	1 nM	0.66 nM	(-)epinephrine (100 µM)	60 min RT	Scintillation counting	249
**β_1 *(h)* (agonist radioligand)**	human recombinant (HEK-293 cells)	[^{3}H](-)CGP 12177	0.3 nM	0.39 nM	alprenolol (50 µM)	60 min RT	Scintillation counting	548
**β_2 *(h)* (antagonist radioligand)**	human recombinant (CHO cells)	[^{3}H](-)CGP 12177	0.3 nM	0.15 nM	alprenolol (50 µM)	120 min RT	Scintillation counting	794
**AT_1 *(h)* (antagonist radioligand)**	human recombinant (HEK-293 cells)	[^{125}I][Sar^1,Ile^8]-AT-II	0.05 nM	0.05 nM	angiotensin-II (10 µM)	120 min 37°C	Scintillation counting	776
**AT_2 *(h)* (agonist radioligand)**	human recombinant (HEK-293 cells)	[^{125}I]CGP 42112A	0.01 nM	0.01 nM	angiotensin-II (1 µM)	4 hr 37°C	Scintillation counting	248
**B_1 *(h)* (agonist radioligand)**	human recombinant (CHO cells)	[^{3}H]desArg^{10}-KD	2 nM	2 nM	desArg^9[Leu^8]-BK (10 µM)	120 min RT	Scintillation counting	525
**B_2 *(h)* (agonist radioligand)**	human recombinant (CHO cells)	[^{3}H]bradykinin	0.3 nM	0.32 nM	bradykinin (1 µM)	60 min RT	Scintillation counting	346
**CB_1 *(h)* (agonist radioligand)**	human recombinant (CHO cells)	[^{3}H]CP 55940	0.5 nM	3.5 nM	WIN 55212-2 (10 µM)	120 min 37°C	Scintillation counting	657
**CB_2 *(h)* (agonist radioligand)**	human recombinant (CHO cells)	[^{3}H]WIN 55212-2	0.8 nM	1.5 nM	WIN 55212-2 (5 µM)	120 min 37°C	Scintillation counting	165
**CCK_1 (CCK_A) *(h)* (agonist radioligand)**	human recombinant (CHO cells)	[^{125}I]CCK-8s	0.08 nM	0.24 nM	CCK-8s (1 µM)	60 min RT	Scintillation counting	562

Table S2. Target binding conditions - continued

Assay	Source	Ligand	Conc.	Kd	Non Specific	Incubation	Detection Method	Bibl.
**CCK_2 (CCK_B) *(h)* (agonist radioligand)**	human recombinant (CHO cells)	[^{125}I]CCK-8s	0.08 nM	0.054 nM	CCK-8s (1 µM)	60 min RT	Scintillation counting	134
**CRF_1 *(h)* (agonist radioligand)**	human recombinant (CHO cells)	[^{125}I]sauvagine	0.075 nM	0.12 nM	sauvagine (0.5 µM)	120 min RT	Scintillation counting	557
**D_1 *(h)* (antagonist radioligand)**	human recombinant (CHO cells)	[^{3}H]SCH 23390	0.3 nM	0.2 nM	SCH 23390 (1 µM)	60 min RT	Scintillation counting	281
**D_{2S} *(h)* (antagonist radioligand)**	human recombinant (HEK-293 cells)	[^{3}H]methyl-spiperone	0.3 nM	0.15 nM	(+)butaclamol (10 µM)	60 min RT	Scintillation counting	87
**D_3 *(h)* (antagonist radioligand)**	human recombinant (CHO cells)	[^{3}H]methyl-spiperone	0.3 nM	0.085 nM	(+)butaclamol (10 µM)	60 min RT	Scintillation counting	145
**$D_{4.4}$ *(h)* (antagonist radioligand)**	human recombinant (CHO cells)	[^{3}H]methyl-spiperone	0.3 nM	0.19 nM	(+)butaclamol (10 µM)	60 min RT	Scintillation counting	252
**ET_A *(h)* (agonist radioligand)**	human recombinant (CHO cells)	[^{125}I]endothelin-1	0.03 nM	0.03 nM	endothelin-1 (100 nM)	120 min 37°C	Scintillation counting	30
**ET_B *(h)* (agonist radioligand)**	human recombinant (CHO cells)	[^{125}I]endothelin-1	0.03 nM	0.04 nM	endothelin-1 (0.1 µM)	120 min 37°C	Scintillation counting	541
**GABA (non-selective) (agonist radioligand)**	rat cerebral cortex	[^{3}H]GABA	10 nM	15 nM	GABA (100 µM)	60 min RT	Scintillation counting	247
**H_1 *(h)* (antagonist radioligand)**	human recombinant (HEK-293 cells)	[^{3}H]pyrilamine	1 nM	1.7 nM	pyrilamine (1 µM)	60 min RT	Scintillation counting	492
**H_2 *(h)* (antagonist radioligand)**	human recombinant (CHO cells)	[^{125}I]APT	0.075 nM	2.9 nM	tiotidine (100 µM)	120 min RT	Scintillation counting	540
**H_3 *(h)* (agonist radioligand)**	human recombinant (CHO cells)	[^{3}H]N$^{\alpha}$-Me-histamine	1 nM	0.32 nM	(R)α-Me-histamine (1 µM)	60 min RT	Scintillation counting	563
**I_2 (antagonist radioligand)**	rat cerebral cortex	[^{3}H]idazoxan (+ 1 µM yohimbine)	2 nM	4 nM	cirazoline (10 µM)	30 min RT	Scintillation counting	27
**$CysLT_1$ (LTD_4) *(h)* (agonist radioligand)**	human recombinant (CHO cells)	[^{3}H]LTD_4	0.3 nM	0.24 nM	LTD_4 (1 µM)	60 min RT	Scintillation counting	618
**MC_4 *(h)* (agonist radioligand)**	human recombinant (CHO cells)	[^{125}I]NDP-α-MSH	0.05 nM	0.54 nM	NDP-α-MSH (1 µM)	120 min 37°C	Scintillation counting	211
**MT_1 (ML_{1A}) *(h)* (agonist radioligand)**	human recombinant (CHO cells)	[^{125}I]2-iodomelatonin	0.01 nM	0.04 nM	melatonin (1 µM)	240 min RT	Scintillation counting	639
**M (non-selective) (antagonist radioligand)**	rat cerebral cortex	[^{3}H]QNB	0.05 nM	0.01 nM	atropine (1 µM)	120 min RT	Scintillation counting	195
**NK_1 *(h)* (agonist radioligand)**	human endogenous (U373MG cells)	[^{125}I]-Substance P LYS3	0.05 nM	0.04 nM	[Sar^9,$Met(O_2)^{11}$]-SP (1 µM)	30 min RT	Scintillation counting	104

Table S2. Target binding conditions - continued

Assay	Source	Ligand	Conc.	Kd	Non Specific	Incubation	Detection Method	Bibl.
**NK_2 *(h)* (agonist radioligand)**	human recombinant (CHO cells)	[^{125}I]NKA	0.1 nM	0.12 nM	[Nleu^{10}]-NKA (4-10) (300 nM)	60 min RT	Scintillation counting	3
**NK_3 *(h)* (antagonist radioligand)**	human recombinant (CHO cells)	[^{3}H]SR 142801	0.4 nM	0.47 nM	SB 222200 (10 µM)	120 min RT	Scintillation counting	741
**Y (non-selective) (agonist radioligand)**	rat cerebral cortex	[^{125}I]peptide YY	0.05 nM	0.1 nM	NPY (1 µM)	120 min RT	Scintillation counting	84
**N neuronal α4β2 *(h)* (agonist radioligand)**	human recombinant (SH-SY5Y cells)	[^{3}H]cytisine	0.6 nM	0.3 nM	nicotine (10 µM)	120 min 4°C	Scintillation counting	1084
**opioid (non-selective) (antagonist radioligand)**	rat cerebral cortex	[^{3}H]naloxone	1 nM	2.6 nM	naloxone (1 µM)	40 min RT	Scintillation counting	43
**NOP (ORL1) *(h)* (agonist radioligand)**	human recombinant (Chem-1 (RBL) cells)	[^{3}H]nociceptin	0.06 nM	0.054 nM	nociceptin (1 µM)	60 min RT	Scintillation counting	1588
**PPARγ *(h)* (agonist radioligand)**	human recombinant (*E. coli*)	[^{3}H]rosiglitazone	5 nM	5.7 nM	rosiglitazone (10 µM)	120 min 4°C	Scintillation counting	567
**EP_2 *(h)* (agonist radioligand)**	human recombinant (HEK-293 cells)	[^{3}H]PGE$_2$	3 nM	3 nM	PGE$_2$ (10 µM)	120 min RT	Scintillation counting	781
**IP (PGI_2) *(h)* (agonist radioligand)**	human recombinant (HEK-293 cells)	[^{3}H]iloprost	6 nM	8 nM	iloprost (10 µM)	60 min RT	Scintillation counting	781
**P2Y (agonist radioligand)**	rat cerebral cortex	[^{35}S]dATPαS	10 nM	10 nM	dATPαS (10 µM)	60 min RT	Scintillation counting	298
**sigma (non-selective) (h) (agonist radioligand)**	human endogenous (Jurkat cells)	[3H]DTG	10 nM	41 nM	Haloperidol (10 µM)	120 min RT	Scintillation counting	1136
**GR *(h)* (agonist radioligand)**	human endogenous (IM-9 cells)	[^{3}H]dexamethasone	1.5 nM	1.5 nM	triamcinolone (10 µM)	6 hr 4°C	Scintillation counting	283
**ER (non-selective) *(h)* (agonist radioligand)**	human endogenous (MCF-7 cells)	[^{3}H]estradiol	0.4 nM	0.2 nM	17-β-estradiol (6 µM)	20 hr 4°C	Scintillation counting	1070
**PR *(h)* (agonist radioligand)**	human endogenous (T47D cells)	[^{3}H]progesterone	0.5 nM	2 nM	promegestone (1 µM)	20 hr 4°C	Scintillation counting	930
**AR *(h)* (agonist radioligand)**	human endogenous (LNCaP cells)	[^{3}H]methyltrienolone	1 nM	0.8 nM	testostérone (1 µM)	24 hr 4°C	Scintillation counting	498
**TRH_1 *(h)* (agonist radioligand)**	human recombinant (CHO cells)	[^{3}H]Me-TRH	2 nM	3.9 nM	TRH (10 µM)	120 min 4°C	Scintillation counting	709
**V_{1a} *(h)* (agonist radioligand)**	human recombinant (CHO cells)	[^{3}H]AVP	0.3 nM	0.5 nM	AVP (1 µM)	60 min RT	Scintillation counting	343
**V_2 *(h)* (agonist radioligand)**	human recombinant (CHO cells)	[^{3}H]AVP	0.3 nM	0.76 nM	AVP (1 µM)	120 min RT	Scintillation counting	343

Table S2. Target binding conditions - continued

Assay	Source	Ligand	Conc.	Kd	Non Specific	Incubation	Detection Method	Bibl.
**Serotonin (5-Hydroxytryptamine) 5-HT1, Non-Selective**	rat cerebral cortex	[3H] Serotonin (5-HT)	1 nM	0.62 nM	Serotonin (5-HT)(10.0 µM)	60 min 25°C	Scintillation counting	1447 , 1567
**Ion channels**								
**BZD (central) (agonist radioligand)**	rat cerebral cortex	[^{3}H]flunitrazepam	0.4 nM	2.1 nM	diazepam (3 µM)	60 min 4°C	Scintillation counting	227
**AMPA (agonist radioligand)**	rat cerebral cortex	[^{3}H]AMPA	8 nM	82 nM	L-glutamate (1 mM)	60 min 4°C	Scintillation counting	166
**kainate (agonist radioligand)**	rat cerebral cortex	[^{3}H]kainic acid	5 nM	19 nM	L-glutamate (1 mM)	60 min 4°C	Scintillation counting	160
**NMDA (antagonist radioligand)**	rat cerebral cortex	[^{3}H]CGP 39653	5 nM	23 nM	L-glutamate (100 µM)	60 min 4°C	Scintillation counting	221
**PCP (antagonist radioligand)**	rat cerebral cortex	[^{3}H]TCP	10 nM	13 nM	MK 801 (10 µM)	120 min 37°C	Scintillation counting	257
**P2X (agonist radioligand)**	rat urinary bladder	[^{3}H]α,β-MeATP	3 nM	2.6 nM	α,β-MeATP (10 µM)	120 min 4°C	Scintillation counting	17
**Ca^{2+} channel (L, dihydropyridine site) (antagonist radioligand)**	rat cerebral cortex	[^{3}H]nitrendipine	0.1 nM	0.18 nM	nitrendipine (1 µM)	90 min RT	Scintillation counting	996
**Ca^{2+} channel (L, diltiazem site) (benzothiazepines) (antagonist radioligand)**	rat cerebral cortex	[^{3}H]diltiazem	15 nM	52 nM	diltiazem (10 µM)	120 min RT	Scintillation counting	212
**Ca^{2+} channel (L, verapamil site) (phenylalkylamine) (antagonist radioligand)**	rat cerebral cortex	[^{3}H]D888	3 nM	3 nM	D 600 (10 µM)	120 min RT	Scintillation counting	194
**K_{ATP} channel (antagonist radioligand)**	rat cerebral cortex	[^{3}H]glibenclamide	0.1 nM	0.05 nM	glibenclamide (1 µM)	60 min RT	Scintillation counting	6
**K_V channel (antagonist radioligand)**	rat cerebral cortex	[^{125}I]α-dendrotoxin	0.01 nM	0.04 nM	α-dendrotoxin (50 nM)	60 min RT	Scintillation counting	225
**SK_{Ca} channel (antagonist radioligand)**	rat cerebral cortex	[^{125}I]apamin	0.007 nM	0.007 nM	apamin (100 nM)	60 min 4°C	Scintillation counting	112
**Na^{+} channel (site 2) (antagonist radioligand)**	rat cerebral cortex	[^{3}H]batrachotoxin	10 nM	91 nM	veratridine (300 µM)	60 min 37°C	Scintillation counting	28
**Cl^{-} channel (GABA-gated) (antagonist radioligand)**	rat cerebral cortex	[^{35}S]TBPS	3 nM	14.6 nM	picrotoxinin (20 µM)	120 min RT	Scintillation counting	136

Table S2. Target binding conditions - continued

Assay	Source	Ligand	Conc.	Kd	Non Specific	Incubation	Detection Method	Bibl.
**Transporters**								
**norepinephrine transporter *(h)* (antagonist radioligand)**	human recombinant (CHO cells)	[^{3}H]nisoxetine	1 nM	2.9 nM	desipramine (1 µM)	120 min 4°C	Scintillation counting	180
**dopamine transporter *(h)* (antagonist radioligand)**	human recombinant (CHO cells)	[^{3}H]BTCP	4 nM	4.5 nM	BTCP (10 µM)	120 min 4°C	Scintillation counting	190
**GABA transporter (antagonist radioligand)**	rat cerebral cortex	[^{3}H]GABA (+ 10 µM isoguvacine) (+ 10 µM baclofen)	10 nM	4600 nM	GABA (1 mM)	30 min RT	Scintillation counting	214
**choline transporter (CHT1) *(h)* (antagonist radioligand)**	human recombinant (CHO cells)	[^{3}H]hemicholinium-3	3 nM	3.9 nM	hemicholinium-3 (10 µM)	60 min RT	Scintillation counting	648
**5-HT transporter *(h)* (antagonist radioligand)**	human recombinant (CHO cells)	[^{3}H]imipramine	2 nM	1.7 nM	imipramine (10 µM)	60 min RT	Scintillation counting	566

Table S3. Target binding of compounds that are reference in Figure S2.

Compound I.D.	IC_{50} (M)	K_i (M)	nH
**A_1 *(h)* (antagonist radioligand)**			
DPCPX	1.4E-09 M	8.7E-10 M	0.9
**A_{2A} *(h)* (agonist radioligand)**			
NECA	3.4E-08 M	2.8E-08 M	1.1
**A_3 *(h)* (agonist radioligand)**			
IB-MECA	4.2E-10 M	2.5E-10 M	0.8
**α_1 (non-selective) (antagonist radioligand)**			
prazosin	3.0E-10 M	7.9E-11 M	0.8
**α_2 (non-selective) (antagonist radioligand)**			
yohimbine	1.0E-07 M	4.1E-08 M	0.9
**β_1 *(h)* (agonist radioligand)**			
atenolol	4.1E-07 M	2.3E-07 M	0.9
**β_2 *(h)* (antagonist radioligand)**			
ICI 118551	8.1E-10 M	2.7E-10 M	1.7
**AT_1 *(h)* (antagonist radioligand)**			
saralasin	9.4E-10 M	4.7E-10 M	0.8
saralasin	1.1E-09 M	5.5E-10 M	1.0
**AT_2 *(h)* (agonist radioligand)**			
angiotensin-II	6.3E-11 M	3.2E-11 M	1.0
**BZD (central) (agonist radioligand)**			
diazepam	9.9E-09 M	8.3E-09 M	0.9
**B_1 *(h)* (agonist radioligand)**			
desArg^{10}-KD	8.5E-10 M	4.3E-10 M	1.0
**B_2 *(h)* (agonist radioligand)**			
NPC 567	8.2E-09 M	4.2E-09 M	0.6
**CB_1 *(h)* (agonist radioligand)**			
CP 55940	9.9E-10 M	8.7E-10 M	0.9
**CB_2 *(h)* (agonist radioligand)**			
WIN 55212-2	2.0E-09 M	1.3E-09 M	0.8
**CCK_1 (CCK_A) *(h)* (agonist radioligand)**			
CCK-8s	9.1E-11 M	6.8E-11 M	1.2
**CCK_2 (CCK_B) *(h)* (agonist radioligand)**			
CCK-8s	2.0E-10 M	8.2E-11 M	0.9
**CRF_1 *(h)* (agonist radioligand)**			
sauvagine	8.4E-10 M	5.2E-10 M	0.7
**D_1 *(h)* (antagonist radioligand)**			
SCH 23390	3.8E-10 M	1.5E-10 M	1.0
**D_{2S} *(h)* (antagonist radioligand)**			
(+)butaclamol	2.7E-09 M	9.1E-10 M	1.2
**D_3 *(h)* (antagonist radioligand)**			
(+)butaclamol	3.0E-09 M	6.6E-10 M	0.8
(+)butaclamol	1.9E-09 M	4.2E-10 M	1.3
**$D_{4.4}$ *(h)* (antagonist radioligand)**			
clozapine	7.1E-08 M	2.8E-08 M	1.3
**ET_A *(h)* (agonist radioligand)**			
endothelin-1	8.6E-11 M	4.3E-11 M	0.9
**ET_B *(h)* (agonist radioligand)**			
endothelin-3	8.4E-11 M	4.8E-11 M	1.2
**GABA (non-selective) (agonist radioligand)**			
GABA	3.6E-08 M	2.2E-08 M	1.2

Table S3. Target binding of compounds that are reference in Figure S2 – continued.

Compound I.D.	IC_{50} (M)	K_i (M)	nH
**AMPA (agonist radioligand)**			
L-glutamate	1.4E-07 M	1.2E-07 M	2.4
L-glutamate	2.7E-07 M	2.5E-07 M	0.8
**kainate (agonist radioligand)**			
kainic acid	2.7E-08 M	2.1E-08 M	0.6
**NMDA (antagonist radioligand)**			
CGS 19755	4.7E-07 M	3.8E-07 M	1.1
**H_1 *(h)* (antagonist radioligand)**			
pyrilamine	2.9E-09 M	1.8E-09 M	1.5
**H_2 *(h)* (antagonist radioligand)**			
cimetidine	7.7E-07 M	7.5E-07 M	1.5
cimetidine	6.9E-07 M	6.7E-07 M	1.1
**H_3 *(h)* (agonist radioligand)**			
(R)α-Me-histamine	1.4E-09 M	3.3E-10 M	1.2
**I_2 (antagonist radioligand)**			
idazoxan	8.8E-09 M	5.9E-09 M	1.0
**$CysLT_1$ (LTD_4) *(h)* (agonist radioligand)**			
LTD_4	6.5E-10 M	2.9E-10 M	1.1
**MC_4 *(h)* (agonist radioligand)**			
NDP-α-MSH	3.0E-10 M	2.7E-10 M	1.2
**MT_1 (ML_{1A}) *(h)* (agonist radioligand)**			
melatonin	1.7E-10 M	1.4E-10 M	1.1
**M (non-selective) (antagonist radioligand)**			
atropine	3.9E-10 M	6.6E-11 M	0.9
**NK_1 *(h)* (agonist radioligand)**			
[Sar^9,$Met(O_2)^{11}$]-SP	4.5E-10 M	2.0E-10 M	0.6
**NK_2 *(h)* (agonist radioligand)**			
[$Nleu^{10}$]-NKA (4-10)	3.3E-09 M	1.8E-09 M	0.7
**NK_3 *(h)* (antagonist radioligand)**			
SB 222200	7.4E-09 M	4.0E-09 M	1.1
**Y (non-selective) (agonist radioligand)**			
NPY	4.5E-10 M	3.0E-10 M	1.2
**N neuronal α4β2 *(h)* (agonist radioligand)**			
nicotine	7.5E-09 M	2.5E-09 M	0.7
**opioid (non-selective) (antagonist radioligand)**			
naloxone	1.9E-09 M	1.4E-09 M	0.8
**NOP (ORL1) *(h)* (agonist radioligand)**			
nociceptin	1.5E-10 M	6.9E-11 M	1.3
**PPARγ *(h)* (agonist radioligand)**			
rosiglitazone	2.0E-08 M	1.1E-08 M	1.3
rosiglitazone	1.8E-08 M	9.6E-09 M	0.9
**PCP (antagonist radioligand)**			
MK 801	9.2E-09 M	5.2E-09 M	0.9
**EP_2 *(h)* (agonist radioligand)**			
PGE_2	1.9E-09 M	9.6E-10 M	1.1
**IP (PGI_2) *(h)* (agonist radioligand)**			
iloprost	2.4E-08 M	1.4E-08 M	0.8
**P2X (agonist radioligand)**			
α,β-MeATP	4.4E-09 M	2.1E-09 M	0.8
**P2Y (agonist radioligand)**			
dATPαS	9.6E-08 M	4.8E-08 M	0.9
**sigma (non-selective) (h) (agonist radioligand)**			
haloperidol	7.0E-08 M	5.7E-08 M	2.2

Table S3. Target binding of compounds that are reference in Figure S2 – further continued

Compound I.D.	IC_{50} (M)	K_i (M)	nH
**GR *(h)* (agonist radioligand)**			
dexamethasone	3.4E-09 M	1.7E-09 M	1.1
**ER (non-selective) *(h)* (agonist radioligand)**			
17-β-estradiol	4.7E-10 M	1.6E-10 M	1.0
**PR *(h)* (agonist radioligand)**			
promegestone	6.9E-10 M	5.5E-10 M	1.6
promegestone	2.9E-10 M	2.4E-10 M	1.3
**AR *(h)* (agonist radioligand)**			
testosterone	3.0E-09 M	1.3E-09 M	0.8
**TRH_1 *(h)* (agonist radioligand)**			
TRH	3.7E-08 M	2.5E-08 M	0.9
**V_{1a} *(h)* (agonist radioligand)**			
$[d(CH_2)_5{}^1,Tyr(Me)_2]$-AVP	2.1E-09 M	1.3E-09 M	1.2
**V_2 *(h)* (agonist radioligand)**			
AVP	1.8E-09 M	1.3E-09 M	1.0
**Ca^{2+} channel (L, dihydropyridine site) (antagonist radioligand)**			
nitrendipine	2.6E-10 M	1.7E-10 M	1.3
**Ca^{2+} channel (L, diltiazem site) (benzothiazepines) (antagonist radioligand)**			
diltiazem	4.8E-08 M	3.7E-08 M	0.5
diltiazem	5.3E-08 M	4.1E-08 M	1.3
**Ca^{2+} channel (L, verapamil site) (phenylalkylamine) (antagonist radioligand)**			
D 600	1.9E-08 M	9.6E-09 M	0.6
**K_{ATP} channel (antagonist radioligand)**			
glibenclamide	1.0E-10 M	3.5E-11 M	0.7
**K_V channel (antagonist radioligand)**			
α-dendrotoxin	3.0E-10 M	2.4E-10 M	>3
**SK_{Ca} channel (antagonist radioligand)**			
apamin	2.5E-11 M	1.3E-11 M	1.5
**Na^+ channel (site 2) (antagonist radioligand)**			
veratridine	2.2E-05 M	2.0E-05 M	1.5
**Cl^- channel (GABA-gated) (antagonist radioligand)**			
picrotoxinin	3.3E-07 M	2.8E-07 M	0.8
**norepinephrine transporter *(h)* (antagonist radioligand)**			
protriptyline	5.4E-09 M	4.0E-09 M	1.2
**dopamine transporter *(h)* (antagonist radioligand)**			
BTCP	9.7E-09 M	5.1E-09 M	2.1
**GABA transporter (antagonist radioligand)**			
nipecotic acid	2.4E-06 M	2.4E-06 M	0.7
**choline transporter (CHT1) *(h)* (antagonist radioligand)**			
hemicholinium-3	1.8E-08 M	1.0E-08 M	0.8
**5-HT transporter *(h)* (antagonist radioligand)**			
imipramine	2.5E-09 M	1.1E-09 M	0.9
**Serotonin (5-Hydroxytryptamine) 5-HT1, Non-Selective**			
Serotonin (5-HT)	2.6E-09 M	1.0E-09 M	0.9

Table S4. Conditions of enzyme and uptake assays.

Assay	Source	Substrate/ Stimulus/Tracer	Incubation	Measured Component	Detection Method	Bibl.
**Epigenetic enzymes and DNA-related enzymes**						
**CENP-E *(h)***	human recombinant (*E. coli*)	ATP (100 µM)	5 min RT	inorganic phosphate	Photometry	853
**Eg5 *(h)***	human recombinant	ATP (50 µM)	10 min RT	inorganic phosphate	Photometry	853
**HDAC3 *(h)***	human recombinant	fluorogenic HDAC substrate (50 µM)	10 min RT	fluoro-lysine	Fluorimetry	896
**HDAC4 *(h)***	human recombinant	fluorogenic HDAC substrate class 2a (20 µM)	30 min RT	fluoro-lysine	Fluorimetry	896
**HDAC6 *(h)***	human recombinant	fluorogenic HDAC substrate (25 µM)	30 min RT	fluoro-lysine	Fluorimetry	896
**HDAC11 *(h)***	human recombinant	fluorogenic HDAC substrate class 2a (50 µM)	30 min 37°C	fluoro-lysine	Fluorimetry	896
**sirtuin 1 *(h)* (inhibitor effect)**	human recombinant (*E. coli*)	fluorogenic HDAC substrate (200 µM)	20 min RT	fluoro-lysine	Fluorimetry	976
**sirtuin 2 *(h)* (inhibitor effect)**	human recombinant (*E. coli*)	fluoro-lysine sirtuin 2 deacetylase substrate (150 µM)	60 min RT	fluoro-lysine	Fluorimetry	976
**Other enzymes**						
**COX1(h)**	human recombinant	Arachidonic acid (3µM) + ADHP (25 µM)	3 min RT	Resorufin (oxydized ADHP)	Fluorimetry	1480
**5-lipoxygenase *(h)***	human recombinant (Sf9 cells) (cytosol)	arachidonic acid (25 µM)	20 min RT	rhodamine 123	Fluorimetry	1068
**PDE1B *(h)***	human recombinant (Sf9 cells)	[3H]cGMP + cGMP (1.5 µM)	20 min RT	[3H]5'GMP	Scintillation counting	1399
**PDE2A1 *(h)***	human recombinant (Sf9 cells)	[3H]cAMP + cAMP (2µM)	20 min RT	[3H]5'AMP	Scintillation counting	1399
**PDE3A *(h)***	human recombinant (Sf9 cells)	[3H]cAMP + cAMP (0.5µM)	20 min RT	[3H]5'AMP	Scintillation counting	1399
**PDE4D2 *(h)***	human recombinant (Sf9 cells)	[3H]cAMP + cAMP (0.5µM)	20 min RT	[3H]5'AMP	Scintillation counting	1399
**PDE5 *(h)* (non-selective)**	human platelets	[^{3}H]cGMP + cGMP (1 µM)	60 min RT	[^{3}H]5'GMP	Scintillation counting	263
**adenylyl cyclase (activator effect)**	CHO cells	none (100 µM forskolin for control)	10 min RT	cAMP	HTRF	1109
**guanylyl cyclase *(h)* (activator effect)**	human recombinant	GTP (10 µM) (100 µM SNP for control)	10 min RT	cGMP	HTRF	1076
**phosphatase 1B *(h)* (PTP1B)**	human recombinant (*E. coli*)	DIFMUP (10 µM)	30 min RT	DIFMU	Fluorimetry	928
**phosphatase CDC25A *(h)***	human recombinant (*E. coli*)	DIFMUP (200 µM)	20 min RT	DIFMU	Fluorimetry	928
**acetylcholinesteras e *(h)***	human recombinant (HEK-293 cells)	Acetylthiocholine (400 µM)	30 min RT	5 thio 2 nitrobenzoic acid	Photometry	63
**GABA transaminase**	rat brain	GABA (9 mM) + α-ketoglutarate (9 mM)	60 min 37°C	succinic semialdehyde	Fluorimetry	286
**MAO-A *(h)***	human placenta	kynuramine (0.15 mM)	20 min RT	4-OHquinoline	Photometry	265

Assay	Source	Substrate/ Stimulus/Tracer	Incubation	Measured Component	Detection Method	Bibl.
**MAO-B *(h)* recombinant enzyme**	human recombinant	D-Luciferin derivative (4 µM)	60 min 37°C	methyl ester luciferin	Luminescence	1134
**tyrosine hydroxylase**	rat striatum	[^{3}H]tyrosine (10 µM)	20 min 37°C	[^{3}H]H_2O	Scintillation counting	168
**ATPase (Na^+/K^+)**	porcine cerebral cortex	ATP (2 mM)	60 min 37°C	Pi	Photometry	71
